# Safety and efficacy of add-on robotic therapy for early mobilization in intermediate neurocritical care: a pilot study

**DOI:** 10.1186/s12984-025-01750-5

**Published:** 2025-10-03

**Authors:** Ann-Kathrin Joerger, Kim K. Peper, Elisabeth R. Jensen, Maria Wostrack, Benedikt Etzig, Nicole Lange, Barbara Vogel, Alexander Koenig, Sami Haddadin, Bernhard Meyer

**Affiliations:** 1 Department of Neurosurgery, Technical University of Munich, TUM University Hospital Rechts der Isar, Ismaningerstr. 22, 81675 Munich, Germany; 2 Munich Institute of Robotics and Machine Intelligence, Technical University of Munich, Georg-Brauchle-Ring 60, 80992 Munich, Germany; 3 Department of Orthopaedics and Sportorthopaedics, Department of Physiotherapy, Technical University of Munich, TUM University Hospital Rechts der Isar, Ismaningerstr. 22, 81675 Munich, Germany; 4 Mohamed Bin Zayed University of Artificial Intelligence, Masdar City, Abu Dhabi, United Arab Emirates

**Keywords:** Early mobilization, Robotic mobilization therapy, Critical care physiotherapy

## Abstract

**Background:**

Early mobilization has become a cornerstone of critical care due to its benefits in mitigating adverse effects associated with prolonged immobility. Individuals with critical neurosurgical conditions face unique challenges for mobilization, including paresis, cognitive dysfunction, and reliance on cerebral monitoring devices. Staffing limitations, high workloads, and person-specific factors further hinder early mobilization. In recent decades, robots have been developed to overcome these barriers. This pilot study aims to evaluate the safety and efficacy of using the VEMOTION^®^ robotic system as an add-on intervention for early mobilization in individuals with critical neurosurgical conditions.

**Methods:**

A randomized controlled pilot study was conducted at a tertiary hospital involving 18 individuals who required intermediate care due to severe neurosurgical conditions. Participants in the control group received standard physiotherapy, while those in the study group received VEMOTION^®^ robot therapy in addition to conventional physiotherapy. The primary outcome was the occurrence of (serious) adverse events (SAEs/AEs), while secondary outcomes included improvements in physical and respiratory function as measured by the Chelsea Critical Care Physical Assessment Tool (CPAx).

**Results:**

No AEs or SAEs were observed in either group related to the therapy. The study group showed greater improvements in the CPAx, with a median increase of 15 (IQR 11–19) points, compared to a median increase of 4 (IQR: 0–5) points in the control group (*p* = 0.0002). In the control group, the median score of the individual items of the CPAx did not change significantly over the course of the therapy, whereas in the study group, the median of each individual item significantly improved over time.

**Conclusions:**

The results of this pilot study indicate that VEMOTION^®^ robotic therapy is a safe and effective adjunct to conventional physiotherapy for the early mobilization of critically ill neurosurgical patients, leading to clinically significant improvements in physical and respiratory function. Further large-scale studies are needed to confirm these findings and establish the robot’s role in daily clinical practice.

**Supplementary Information:**

The online version contains supplementary material available at 10.1186/s12984-025-01750-5.

## Introduction

Early mobilization therapy, which involves initiating physical activity in individuals who are critically ill in the intensive care unit (ICU) and intermediate care unit (IMC), has become a fundamental component of modern critical care management over the past decades [1]. Individuals in the ICU require the highest level of monitoring, care, and life support. They are often dependent on invasive monitoring techniques and advanced life-sustaining therapies such as mechanical ventilation or continuous administration of catecholamines. Care in the ICU typically involves a very high nurse-to-patient ratio to ensure continuous observation and rapid intervention. Individuals in the IMC, by contrast, are those whose conditions are more severe than can be safely managed on a general ward, but who do not require the full scope of ICU-level care. They require close monitoring and frequent nursing attention, but they are typically not dependent on mechanical ventilation and usually do not require continuous infusions of catecholamines. IMC units serve as a step-up from general wards or a step-down from intensive care.

Prolonged immobility is associated with numerous adverse effects, including muscle atrophy, weakness, restricted joint mobility, critical illness polyneuropathy, pressure ulcers, ventilator-associated pneumonia, an increased risk of thromboembolic events, cognitive impairment, and psychological disorders [2–4]. Numerous studies have investigated early mobilization, reporting mixed findings, with some studies demonstrating beneficial effects, while others have shown contradictory or inconclusive results. Among those reporting positive outcomes, early mobilization has been shown to significantly reduce ICU-acquired weakness, shorten ICU and hospital length of stay, decrease the duration of delirium, and improve the Medical Research Council (MRC) score and Barthel Index at discharge [5–7]. Additionally, it has been shown to lower the incidence of deep vein thrombosis, ventilator-associated pneumonia, and pressure ulcers [5]. However, early mobilization does not appear to reduce ICU mortality rates or the duration of mechanical ventilation [5]. Recent results from the Treatment of Early Activity and Mobilization (TEAM) trial showed that increased very early (i.e., a median of 60 h after ICU admission) active mobilization for individuals receiving mechanical ventilation in the ICU did not lead to a significantly higher number of days alive and out of the hospital compared to the standard mobilization practice [8]. Additionally, active mobilization was associated with a higher incidence of Adverse Events (AEs), such as cardiovascular instability, desaturation, and medical device dislocation. These findings indicate that early mobilization may not be appropriate for all individuals in the ICU and suggest that the timing of initiation of mobilization may also play a critical role, emphasizing the importance of a thorough screening process for appropriate candidates.

Individuals with severe neurological or neurosurgical conditions requiring ICU or IMC care constitute a unique population, as they often experience autonomic dysregulation, including arrhythmias and hypo- or hypertension [2]. They may also exhibit impaired cerebral autoregulation, cognitive dysfunction, and hemiparesis, and are frequently dependent on cerebral monitoring devices [9]. These factors can compromise individual safety during mobilization and may complicate the implementation of early mobilization protocols. However, despite these potential barriers, a growing body of evidence suggests that early mobilization in neurocritical care settings is not only feasible but also safe and beneficial. Several studies have demonstrated positive effects of early mobilization after stroke or severe brain injury in neuro-ICU and neuro-IMC settings [10–12].

There is no strict definition of early mobilization. It comprises bedside exercises, transitioning tasks, passive- and active-assisted range-of-motion exercises, breathing exercises, manual techniques, and sitting, standing, and gait training [2, 3] tailored to the individual’s specific condition and abilities. These standard methods can be supplemented with electrical muscle stimulation, ergometer training, and tilt-table therapy [2].

Barriers to early mobilization include staffing limitations, high workloads, time constraints, and person-specific factors such as obesity, reduced consciousness, and dependence on medical devices [3]. In recent decades, robots have been developed to overcome these barriers. They help to reduce the physical strain on healthcare professionals by automating parts of the mobilization process, such as lifting or moving individuals. For example, Brinkmann et al. [13] demonstrated that using a supportive robotic arm during individual repositioning significantly reduced the physical strain on nursing staff. The VEMOTION^®^ (Reactive Robotics, Munich, Germany) robot assists in verticalizing individuals in their beds and in generating leg movements. Robotic systems not only help to overcome the environmental barriers to mobilization but also offer several distinct advantages for rehabilitation. Among the primary benefits, robotic therapy enables consistent, high-intensity training over extended periods, supporting the delivery of standardized rehabilitation protocols [14]—an essential prerequisite for promoting neuroplasticity. Robotic devices are equipped with sensors that monitor the performance in real time and adapt movement trajectories and assistance levels accordingly [14]. A common feature of many robotic systems is their ability to partially or fully support body weight and facilitate limb movement, which enables individuals to engage in gait training at higher intensities while reducing energy expenditure and cardiorespiratory strain [15]. Moreover, robotic devices can alleviate many of the labor-intensive aspects of conventional physical therapy. When robotic therapy is implemented as an adjunct to conventional physiotherapy, this allows physiotherapists to focus more on individualized, functional rehabilitation during conventional sessions, while supervising multiple individuals during robot-assisted training [15]. Another advantage is the objective quantification of performance-related metrics, such as range of motion, movement velocity, smoothness, and applied force, which can be used to monitor compliance and progress over time [14, 15].

Secondary benefits include increased participant motivation, which is often enhanced through the integration of gamified elements and real-time feedback. In robot-assisted therapy, serious gaming scenarios can provide extrinsic feedback, with performance scores that help maintain engagement and promote adherence to the rehabilitation program [15]. In addition, regularly reviewing the recorded performance data can reinforce the importance of exercise in the participant’s mind and further encourage continued participation, thereby strengthening motivation and adherence [16].

Despite the apparent advantages, robotic systems have not yet been integrated into clinical practice, and scientifically reliable data from clinical settings are scarce. A case study has demonstrated that using VEMOTION^®^ led to an increase in active muscle contraction of an individual in the ICU with critical illness polyneuropathy over the study period [17]. Furthermore, the individual’s electromyographic (EMG) pattern began to approximate that of healthy controls. However, these findings only suggest a potential benefit based on EMG measurements. A clinical benefit for individuals and the safety of its application have not yet been scientifically established. The present pilot study investigates the safety and efficacy of an add-on robotic therapy with the VEMOTION^Ⓡ^ device for early mobilization of individuals with critical neurosurgical conditions compared to conservative physiotherapy alone.

## Methods

### Study design

We conducted a randomized controlled prospective study in a neurosurgical department at a tertiary hospital from 1 August 2023 until 31 March 2025. The study was designed as a pilot study comprising 30 individuals. Individuals with a severe neurosurgical condition that prevented independent mobilization out of bed (e.g., sepsis associated with spondylodiscitis, severe traumatic brain injury) and required intermediate care were included in the study. Additionally, participants had to be sufficiently conscious to follow simple commands. To ensure that all participants were sufficiently alert and conscious, the Richmond Agitation-Sedation Scale (RASS) was assessed for every individual screened. Only those with a RASS score of ≥ 0 were included in the study. In cases of severe traumatic brain injury (TBI), individuals were only considered for inclusion once an adequate level of consciousness had been reached, meaning that study participation was intentionally initiated at a later stage of their hospital stay. Individuals with aneurysmal subarachnoid hemorrhage, stroke, intracranial neoplasia, and paresis or paralysis were excluded. Detailed inclusion and exclusion criteria are provided in Table 1.

**Table 1:** Inclusion and exclusion criteria

Inclusion criteria	≥ 18 years old Immobility corresponding to CPAx level ≤ 1 for the item “Walking steps” Ability to follow commands (at least partially) Height: 1.50 m < *n* < 1.95 m Weight: 45 kg < *n* < 135 kg Stable cardiopulmonary condition allowing for mobilization (see exclusion criteria below) RASS ≥ 0
Exclusion criteria	< 18 years old CPAx level > 1 for the item “Walking steps” Pregnancy Reduced consciousness preventing the ability to follow commands RASS < 0 Aneurysmal subarachnoid hemorrhage Intracranial neoplasia Acute stroke Paresis/Paralysis Cardiopulmonary instability (systolic BP > 180 mmHg / < 80 mmHg, MAP > 110 mmHg / < 65 mmHg, SpO_2_ < 90%, HR > 130 bpm / < 60 bpm, respiratory rate > 30/min / < 10/min) Acute elevated intracranial pressure (within the last 7 days) Pacemaker / ICD Status epilepticus Acute intoxication Shock requiring catecholamine dosage > 0.3 µg/kg/min General catecholamine dosage > 0.3 µg/kg/min i.v. antihypertensive medication Clinical signs of volume overload (massive edema, B-lines on lung ultrasound) BiPAP ventilation New arrhythmias Active bleeding Threatening organ rupture Multi-organ failure with lactate > 4 mmol/l Prescribed bed rest or contraindication for weight-bearing on the spine or lower extremities Severe skin damage at areas in contact with the device Fasciitis, rhabdomyolysis PAD Stage IV Palliative condition with life expectancy < 7 days Dialysis catheter in the groin

BiPAP = Bilevel Positive Airway Pressure, BP = blood pressure, CPAx = Chelsea Critical Care Physical Assessment tool, HR = heart rate, ICD = Implantable Cardioverter Defibrillator Disease, MAP = Mean Arterial Pressure, PAD = Peripheral Artery Disease, RASS = Richmond Agitation-Sedation Scale, SpO_2_ = Peripheral Capillary Oxygen Saturation

All individuals were screened for eligibility based on the predefined inclusion and exclusion criteria by a board-certified neurosurgeon (AKJ). This physician also conducted recruitment, enrollment, and randomization of participants. Importantly, the same physician was blinded to outcome parameter assessment, but not to the statistical analysis and interpretation of the results.

In the context of the present study, “early mobilization“ was not strictly defined by a specific time frame. Rather, it referred to the early phase of mobilization initiated once individuals had achieved cardiopulmonary stability after experiencing a severe neurosurgical condition. This concept differs from mobilization efforts typically undertaken during the rehabilitation phase, as our focus was on initiating activity during the acute recovery period while participants were still hospitalized in the acute care setting.

After enrollment in the study, participants were randomly assigned to a group. Randomization was performed by block randomization: To establish the robotic method in the clinical routine, the first ten participants were block-randomized into the study group, followed by alternating allocation to the control and study group. The control group received conventional physiotherapy according to the hospital’s standard, once daily for 14 days. A standard care session consisted of 15 min of therapy. Exercises were tailored to each individual’s condition. At the lowest level of participation, passive joint mobilization was performed, starting with single peripheral joints and gradually progressing to the entire extremity. This was carried out using the Proprioceptive Neuromuscular Facilitation (PNF) approach. At a more active participation level, mobilization was performed with manual resistance. The highest level of participation included mobilization to the edge of the bed, assisted standing, and assisted walking. In addition, respiratory training was conducted. For participants with the lowest level of activity, this was provided passively through positioning techniques. For those with a higher level of participation, breathing techniques were used to reduce pain or to initiate movement. All exercises were accompanied by verbal communication to help de-escalate agitation or distress.

The study group received VEMOTION^®^ robot therapy consisting of verticalization and cyclic leg movement in addition to conventional physiotherapy once daily for 15 min for 14 days. The net therapy time with the robot began once the leg movement started.

The study protocol determined that if therapy could not take place on a given day for various reasons (e.g., surgery day, scheduled examinations, etc.), that day would be added at the end of the study period. However, a maximum of three days could be made up. The study protocol also accounted for the fact that, in clinical practice, participants may need to be transferred to rehabilitation earlier than planned. The protocol was designed to allow for early transfer, i.e., early termination of study therapy, to ensure that the study did not interfere with the rehabilitation process for ethical reasons. In such cases, a maximum of three days was also allowed.

### VEMOTION^®^ robot

The VEMOTION^®^ robot (Reactive Robotics GmbH, Munich, Germany) was developed for the early mobilization phase in individuals who still require intensive or intermediate care. The bed is modified to be verticalizable, and the robot can be directly connected to the individual’s bed for therapy sessions, allowing for exercise in an upright position within the bed, thereby eliminating the need for a resource-intensive transfer. Adapters attached to the knee joint enable the passive movement of the individual’s legs. During leg movement, the bed can be adjusted to a vertical position up to 70° depending on the individual’s comfort. To ensure stability, individuals are connected to the robot at their feet and secured to the bed using a specially designed chest and seat belt system, preventing forward tipping or collapsing. The criteria for discontinuation of therapy were as follows: systolic blood pressure (RRsys) > 180 mmHg / < 80 mmHg, SpO2 < 90%, heart rate (HR) > 130 bpm / < 60 bpm, HR increase > 20%, new arrhythmias, respiratory rate > 30/min / < 10/min, requirement for resuscitation, deterioration in vigilance/agitation/(suspected) pain (Numeric Rating Scale > 3 or Behavioral Pain Scale > 6), dislocation of access points/catheters/drains and vomiting/reflux. Participants in the robotic therapy group were under continuous monitoring during the therapy session for blood pressure, electrocardiogram, heart rate, SpO₂, and temperature.

The robot features both a passive mode and an assist-as-needed (AAN) function, which adjusts the level of mechanical support based on the individual’s ability to activate their muscles. The AAN function is based on machine learning [18].

### Outcome parameters

The primary endpoint was the recording of Adverse Events (AEs) and Serious Adverse Events (SAEs) in both groups related to the respective therapy. Secondary endpoints included the improvement in the Chelsea Critical Care Physical Assessment Tool (CPAx) at the beginning and end of therapy and changes in the Barthel Index at discharge and three months after discharge from the hospital.

### Adverse events (AEs) and serious adverse events (SAEs)

An AE was defined as any unfavorable and unintended sign or symptom that occurred in temporal association with the use of the robotic device, including, in particular, the predefined criteria for therapy discontinuation (Supplementary Table 1). A SAE was defined as any reaction related to the robotic therapy that resulted in death, was life-threatening, prolonged the hospital stay, or resulted in persistent or significant disability.

### Barthel index (BI)

The Barthel Index is a well-established, simple index to measure a person’s ability to perform activities of daily living (ADLs) [19, 20]. The scale comprises ten items: feeding, bathing, grooming, dressing, bowel control, bladder control, toilet use, transfers, mobility on level surfaces, and stair use. Each item is scored in 5-point increments. The total score ranges from 0 to 100, with lower scores indicating greater levels of dependence. An advantage of the scale is its ease of use; however, a limitation is its lack of sensitivity at the lower and upper ends of the scale, commonly referred to as floor and ceiling effects.

### Declaration of generative AI and AI-assisted technologies in the writing process

While preparing this work, the authors used ChatGPT3.5 (OpenAI, San Francisco, California, USA) and Grammarly (Grammarly, Inc., San Francisco, California, USA) to check spelling, grammar, and style. After using this service, the authors reviewed and edited the content as needed. They take full responsibility for the publication’s content.

### Statistics

To compare the groups with respect to categorical variables, such as gender and primary diagnosis, Fisher’s exact test was used. Given the small sample size, the Mann-Whitney U test was applied to compare the groups with respect to age, the change in CPAx from the beginning to the end of the study, changes in the Barthel Index from discharge to three months post-discharge, length of hospital stay, time to study initiation, and duration of ICU stay. To compare the progression of the scores of individual CPAx items over time and the progression of verticalization, a mixed-effects analysis was performed for each item. A p-value of < 0.05 was considered statistically significant. Statistical analysis was performed with GraphPad Prism 10.3.1 (La Jolla, California, USA).

To examine the influence of group assignment and Barthel Index at discharge on Barthel Index at 3-month follow-up, an analysis of covariance (ANCOVA) was performed. This analysis was conducted using DATAtab (DATAtab Team, 2023). DATAtab: Online Statistics Calculator. DATAtab e.U., Graz, Austria. Available at: https://datatab.de/.

## Results

### Participant overview

**Fig. 1:**
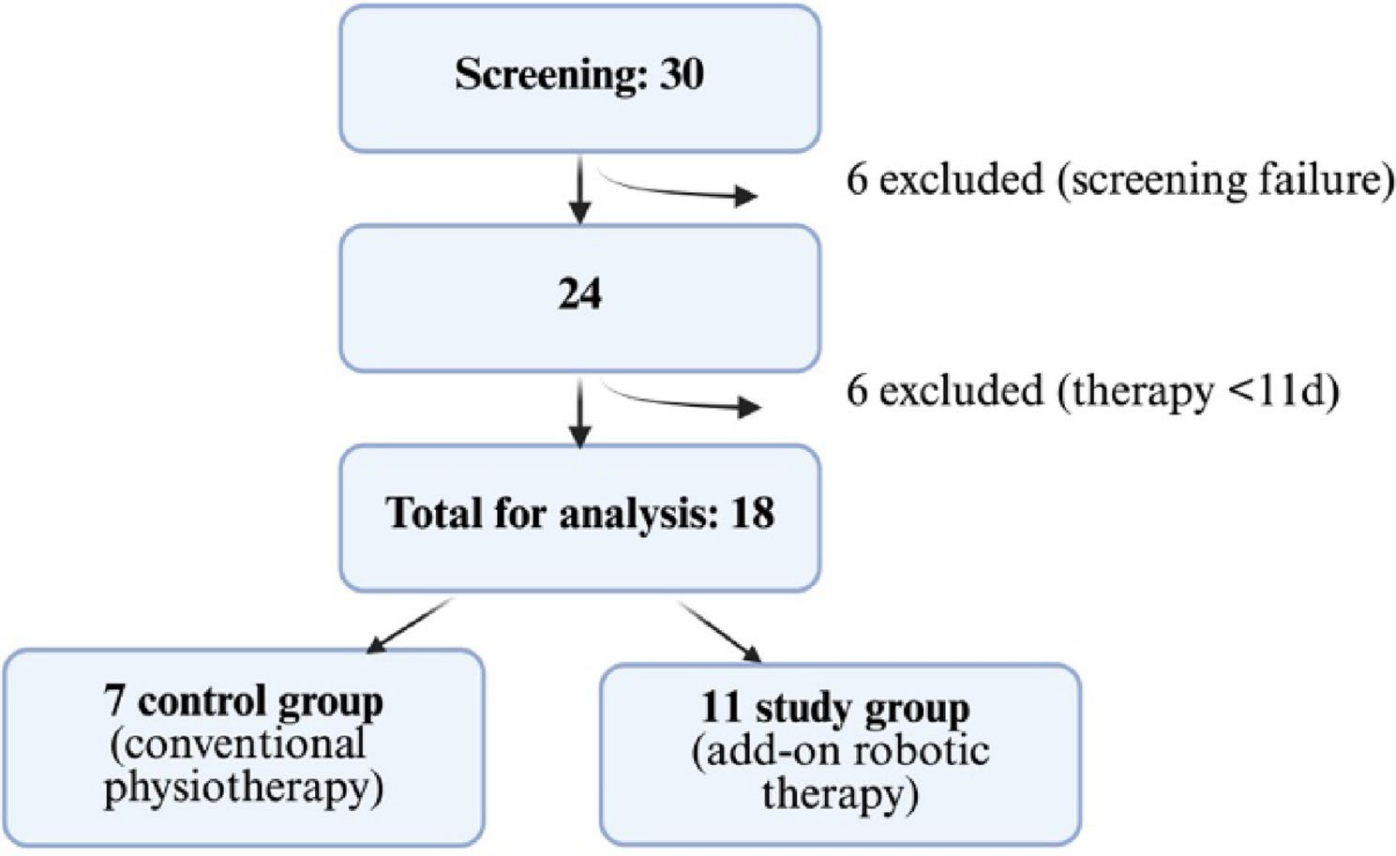
Provides an overview of the participants. Created in BioRender. Joerger, A. (2025) https://BioRender.com/mwuhgn1

From 1 August 2023 until 31 March 2025, 30 individuals were screened for inclusion in the study. Six did not meet the inclusion and exclusion criteria and were excluded as early dropouts (Fig. 1). Of these six early dropouts, four individuals were excluded because they scored too high on the initial CPAx item “stepping” at baseline. One individual was excluded due to an acute episode of delirium shortly after study inclusion, which made them uncooperative and unable to continue participation. One individual withdrew consent shortly after enrollment. An additional six individuals were excluded from the analysis because they received treatment for less than eleven days: three were prematurely transferred to follow-up rehabilitation, two experienced severe clinical deterioration unrelated to the therapy that precluded further participation, and one withdrew consent to participate in the study. Finally, a total of 18 individuals with a median age of 81 (min. – max.: 42–90) years were included in the analysis. Eleven participants were randomized to add-on robot therapy (study group) and seven to standard physiotherapy (control group).

Study-specific therapy commenced at a median of 21 (IQR: 12–38) days after admission to the hospital in the study group and at a median of 14 (IQR: 9–28) days in the control group (*p* = 0.7744) (Table 2). Median total hospital stay and ICU stay did not differ significantly between the groups (Table 2). The groups were comparable in terms of age and sex distribution (Table 2). In the study group, cranial conditions were more common the admission diagnosis than spinal conditions, whereas the control group showed an approximately equal distribution (Table 2).

**Table 2:** Participant overview

	Study group *n* = 11	Control group *n* = 7	
Age median (min -max)	75 (42–90)	83 (75–89)	*p* = 0.1841
Sex	7 (63.6)	3 (42.9)	*p* = 0.6305
Male n (%)			
Female n (%)	4 (36.4)	4 (57.1)	
Admission diagnosis			
Cranial n (%)	8 (72.7)	3 (42.9)	*p* = 0.3322
Spinal n (%)	3 (27.3)	4 (57.1)	
Days in hospital to study start median (IQR)	21 (12–38)	14 (9–28)	*p* = 0.7744
Days in hospital median (IQR)	37 (28–63)	27 (23–49)	*p* = 0.4117
	*n* = 7	*n* = 3	
Days in ICU median (IQR)	20 (11–27)	26 (18–81)	*p* = 0.3833

Sex and admission diagnosis: Fisher’s exact test; age, days in hospital, days to study start, and days in ICU: Mann-Whitney U test; *p* < 0.05 = statistically significant

Detailed admission diagnoses and details of medical treatment can be found in Supplementary Table 2. It is particularly noteworthy that six out of eleven individuals (54.5%) in the study group and two out of seven individuals (28.6%) in the control group had an external ventricular drain in place during the study period (Supplementary Table 2). In the study group, 8 out of 11 participants (72.7%) were transferred directly to rehabilitation facilities, 2 out of 11 (18.2%) were transferred to another hospital, and 1 out of 11 (9.1%) was discharged to a nursing home. In the control group, 4 out of 7 participants (57.1%) were transferred to rehabilitation, 2 out of 7 (28.6%) to a nursing home, and 1 out of 7 (14.3%) was discharged home (Supplementary Table 2).

### Adverse events and serious adverse events

Neither in the study group nor in the control group did any Adverse Event (AE) or Serious Adverse Event (SAE) occur related to the applied study-specific therapy. Furthermore, the therapy did not need to be discontinued or interrupted at any point based on the predefined discontinuation criteria because of clinical deterioration in relation to the study-specific therapy. However, three participants deteriorated clinically independently from the study therapy and were transferred to the ICU acutely: One participant in the control group with known heart failure and severe spondylodiscitis experienced cardiac decompensation in the context of the infection three days after study enrollment. Due to multi-organ failure resulting from severe spondylodiscitis, the treatment approach was shifted to a palliative concept in accordance with the participant’s previously expressed wishes, and the participant subsequently passed away. Another participant in the control group suffered from spontaneous intraventricular bleeding due to anticoagulant therapy nine days after study enrollment and underwent emergency surgery, after which they were transferred to the ICU. Both were excluded from the final analysis because the treatment duration was only three and nine days, respectively. Another participant in the control group was transferred to the ICU because of respiratory deterioration due to aspiration pneumonia, eleven days after study enrollment. They were included in the final analysis.

### Therapy details and CPAx

**Fig. 2:**
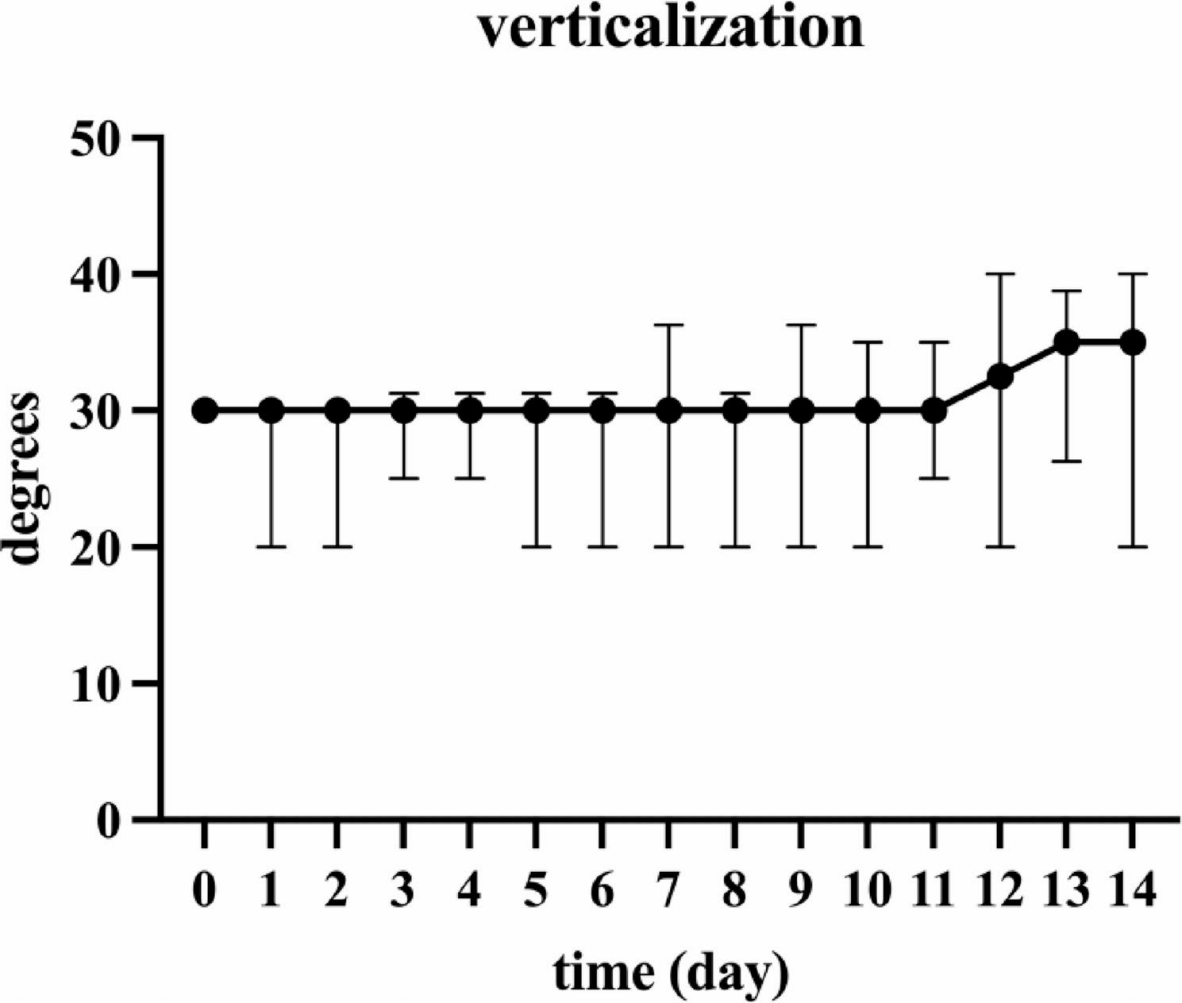
Illustrates the increase in bed verticalization over time during VEMOTION^®^ therapy. The data are presented as the median with interquartile range (IQR) for the 11 participants in the study group.* P*= 0.1177 (mixed-effects analysis)

In the study group, depending on the participant’s performance, the bed in which the robotic therapy took place was manually adjusted for verticalization, and the angle was recorded during each session. The median verticalization for all individuals in the study group was 30 (IQR 20–30) degrees at the beginning of the study and 35 (IQR 20–40) degrees at the end of the study (*p* = 0.1177) (Fig. 2).

**Fig. 3:**
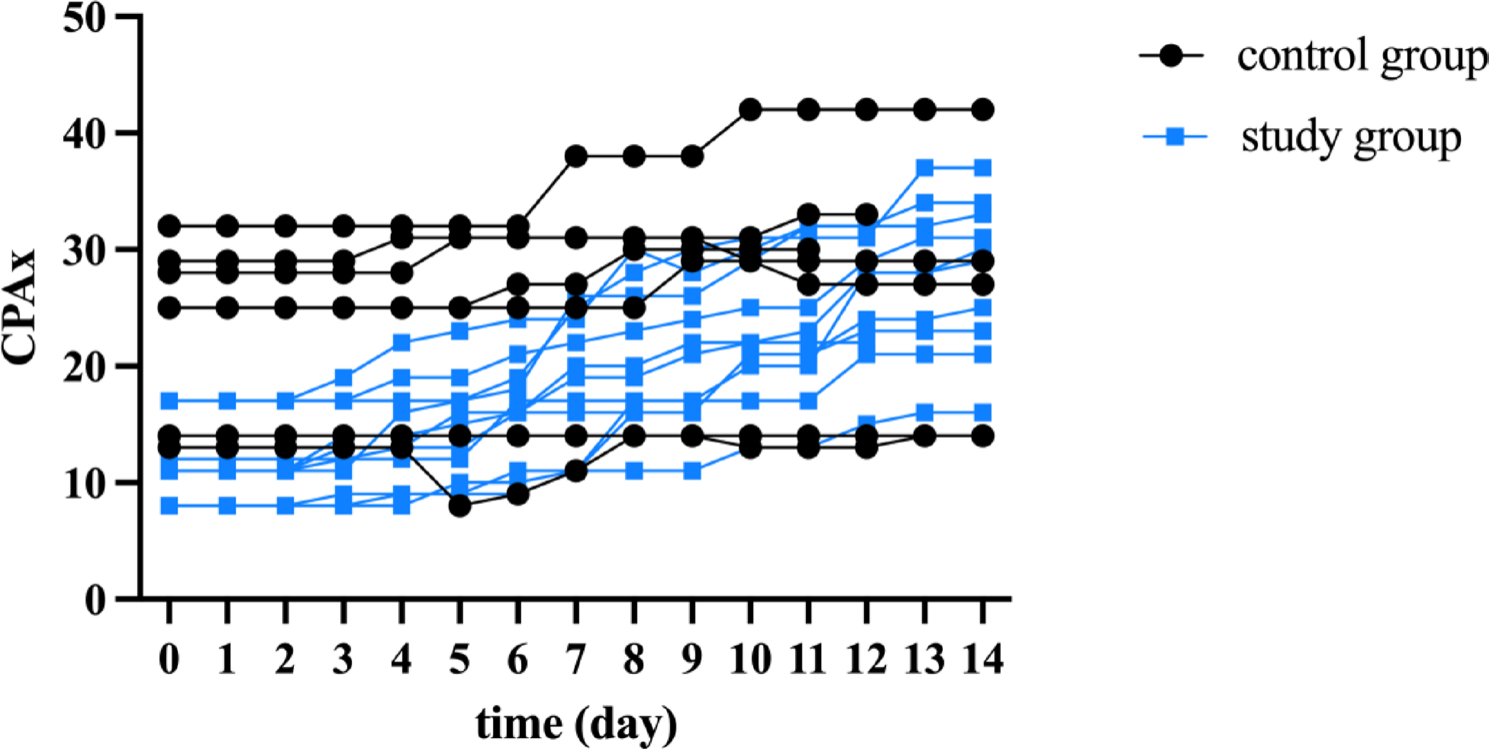
Shows the temporal course of CPAx during the study for each individual in both the study and control group.

In the study group, the CPAx improved for all eleven participants (Fig. 3). In contrast, in the control group, only five out of seven individuals showed an improvement; for one individual, the score remained stable throughout the study period, while for another individual, it decreased by two points.

**Fig. 4:**
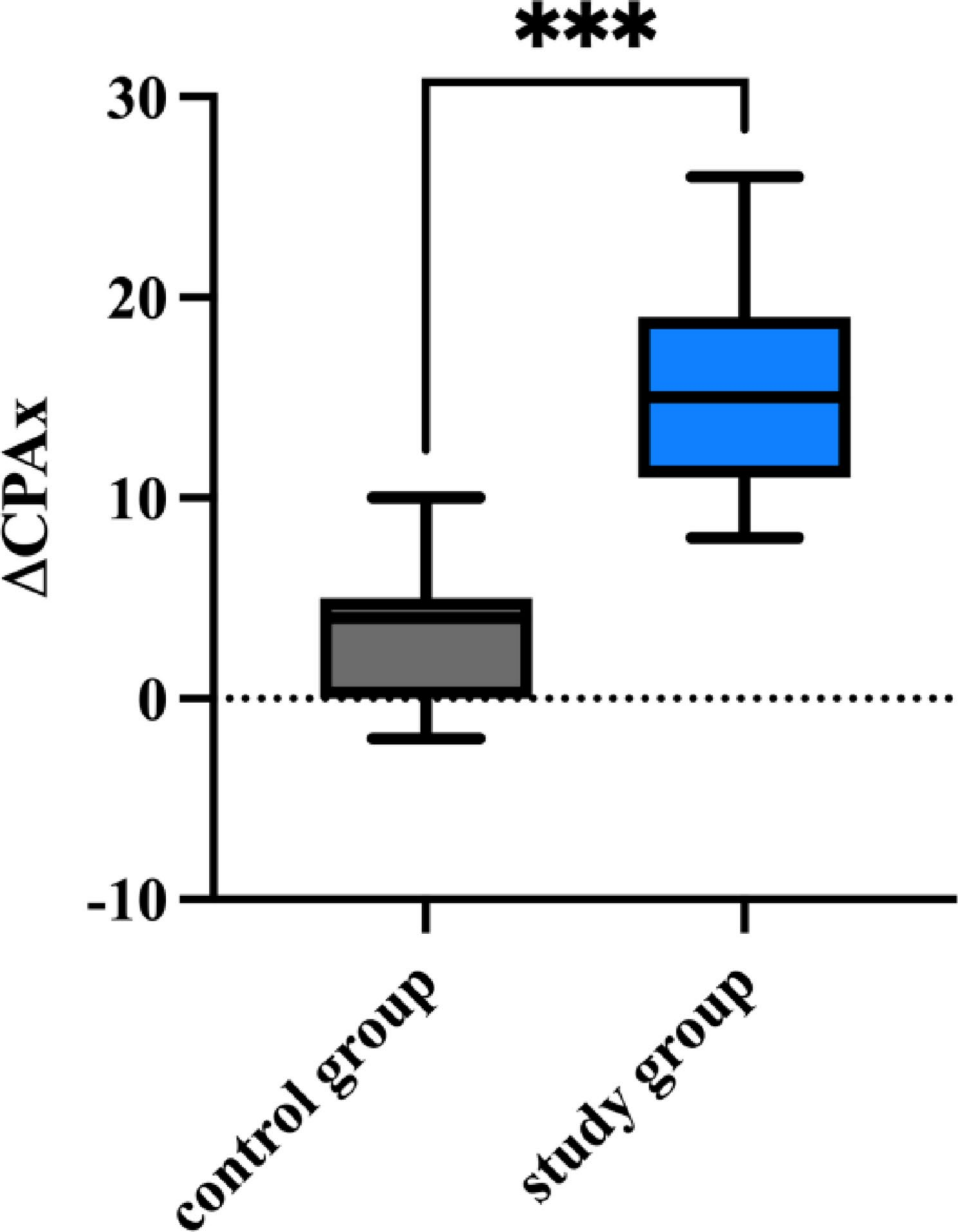
Compares the change in CPAx from the beginning to the end of the study (distributions presented as box plots) for the study and control groups. P= 0.0002

The CPAx improved by a median of 15 (IQR 11–19) points in the study group, whereas it only increased by a median of 4 (IQR: 0–5) points in the control group (*p* = 0.0002) (Figs. 3 and 4)

In the study group, the largest increase in the CPAx was observed on days ten and twelve (median increase 2, IQR d10: 1–3 and IQR d12: 0–4), whereas, in the control group, the median CPAx did not increase on any day. In the control group, the median score of the individual items of the CPAx did not change significantly over the course of the therapy (Supplementary Fig. 1A – J), whereas in the study group, the median of each individual item significantly improved over time (Supplementary Fig. 2A – J). Among all individuals in the study group, the largest changes were observed in the items “moving within the bed”, “dynamic sitting”, and “grip strength” (each with a median of 2, IQR: 1–3, respectively).

### Follow-up and Barthel index

Three-month follow-up data were available for twelve out of eighteen participants (66.7%): five in the control group and seven in the study group. The missing follow-ups were due to the fact that these participants had passed away in the meantime. In the study group, four individuals were still undergoing follow-up rehabilitation, two individuals had been discharged home, one individual remained in a nursing care center, and four individuals had died in the interim. In the control group, two individuals were still undergoing follow-up rehabilitation, three individuals had been discharged home, and two individuals had died in the interim. The median Barthel Index in the study group improved significantly from 10 (IQR: 10–20) at hospital discharge to 55 (IQR: 40–65) at three months (*p* = 0.0156), while in the control group, the median Barthel Index remained unchanged at 15, with an IQR of 5–30 at discharge and 10–50 at three months (Table 3).

Analysis of Covariance (ANCOVA) revealed a significant effect of group assignment on the Barthel Index three months after hospital discharge (F (1, 9) = 5.14, *p* = 0.050), indicating a meaningful difference between the study and control groups (Supplementary Table 3). In contrast, the Barthel Index at discharge did not significantly influence the follow-up score (F (1, 9) = 0.12, *p* = 0.734), suggesting that functional status at discharge had minimal impact on the Barthel Index at three months.

**Table 3:** Differences in Barthel index 3 months after discharge

Group/Participant	Barthel Index Discharge	Barthel Index after 3 months	Δ Barthel Index	BI Discharge vs. BI 3 months
**Study group**				
No. 1	35	55	20	* *p* = 0.0156
No. 5	20	45	25	
No. 6	10	n.a.	n.a.	
No. 7	10	35	25	
No. 8	10	40	30	
No. 10	0	65	65	
No. 11	15	n.a.	n.a.	
No. 14	35	65	30	
No. 16	10	n.a.	n.a.	
No. 20	10	n.a.	n.a.	
No. 23	10	75	65	
Median (IQR)	10 (10–20)	55 (40–65)	30 (25–65)	
**Control group**				*p* > 0.9999
No. 2	5	5	0	
No. 12	0	70	70	
No. 15	30	30	0	
No. 21	15	15	0	
No. 24	20	n.a.	n.a.	
No. 27	15	15	0	
No. 28	35	n.a.	n.a.	
Median (IQR)	15 (5–30)	15 (10–50)	0 (0–35)	
Study vs. control group	*p* = 0.6349	*p* = 0.0644	*p* = 0.0745	

Normality was assessed using the Shapiro-Wilk test. The Barthel Index (BI) at discharge in the study group did not follow a normal distribution. In addition, the sample size of the control group at follow-up (*n* = 5) was too small to reliably test for normality. Therefore, non-parametric tests were used throughout.

Between-group comparisons (BI at discharge, at 3 months, and Δ BI) were performed using the Mann-Whitney U test.

Within-group comparisons (BI at discharge vs. BI at 3 months for study group and control group, respectively) were analyzed using the Wilcoxon signed-rank test.

## Discussion

This is the first randomized controlled pilot study demonstrating the safety and efficacy of an add-on robot therapy for early mobilization of individuals with critical neurosurgical conditions treated in an intermediate care (IMC) unit, as compared to individuals receiving conservative physiotherapy alone.

###  Adverse Events and Serious Adverse Events

In this study, no Adverse Events (AEs) or Serious Adverse Events (SAEs), or interruptions of the running therapy related to the study-specific therapy were observed - a rate even lower than the 1.8% AE rate reported by Warmbein et al. [21], who also applied VEMOTION^®^ robotic therapy to 16 postoperative intensive care individuals. In our study, the median degree of bed verticalization could be slightly increased over the course of time, suggesting good tolerance among participants. It is particularly noteworthy that in the study group, more than half of the individuals had an external ventricular drain (EVD) in place during the robotic therapy. This type of external drain poses a specific risk of dislocation during mobilization. However, no dislocations occurred. These findings suggest that VEMOTION^Ⓡ^ robotic mobilization therapy can be safely implemented in clinical practice, even among individuals with complex monitoring needs, access lines, and drain systems.

### Improvement of physical and respiratory function

This study demonstrated that an add-on robotic therapy significantly improved the physical and respiratory function of individuals with critical neurosurgical conditions, as evidenced by a significantly greater increase in the CPAx in the study group compared to the standard physiotherapy group. Not only did the total score improve more substantially, but each individual item within the score showed significant enhancement. To date, the only study offering a reference point for a minimally clinically important difference (MCID) of the CPAx is a study by Corner et al., which defined a 6-point change as meaningful in a population recovering from severe burn injuries [22]. Using this threshold, our study was even able to demonstrate that the robot-assisted therapy group achieved a clinically relevant improvement in the total CPAx, whereas the control group did not reach this benchmark. However, the only notable similarity between the individuals in the study by Corner et al. [22] and those in our cohort is the severity of illness, in that both groups required care beyond standard ward-level treatment (i.e., intensive or intermediate care). Given the substantial differences in underlying diagnoses, functional impairments, and recovery trajectories, the applicability of the MCID established by Corner et al. to our patient population must be approached with caution and remains questionable. Further research is needed to define population-specific MCID thresholds for CPAx in neurosurgical critically ill patients.

A study most comparable to ours was conducted by Huebner et al. [23], who enrolled 16 individuals following lung transplantation in the ICU. Participants received twice-daily VEMOTION^®^ robotic therapy for one week immediately after surgery, and were compared to a retrospective control group of 13 conventionally mobilized individuals. No significant differences were observed in ventilation duration, ICU stay, muscle ultrasound, or quality of life at three months post-surgery. However, their outcome parameters differed entirely from ours, and except for the muscle ultrasound measures, none of their assessed parameters reflected the individual’s physical function.

Calabrò et al. [24] evaluated Erigo^®^ robotic therapy versus physiotherapist-assisted verticalization therapy in 20 individuals after stroke. Outcomes included lower limb motor function, degree of paresis, postural control, cognitive function, vestibular function, and cortical excitability. Like VEMOTION^Ⓡ^, Erigo^Ⓡ^ integrates verticalization and passive or active-assisted, step-like leg movements, though the latter requires individual transfer to a tilt table. In contrast, VEMOTION^Ⓡ^ enables therapy in the individual’s bed. Calabrò et al. [24] reported significantly greater improvements with robotic therapy compared to physiotherapist-assisted verticalization therapy, particularly in lower limb motor function, postural control, and cognitive abilities. Although these authors examined individual aspects of physical function, their study, unlike the present one, does not allow for any conclusions regarding overall mobility.

Another pilot study by Calabrò’s group [25] demonstrated that combining music therapy with robotic verticalization in individuals in a minimally conscious state due to traumatic brain injury led to improvements in cognitive function, level of consciousness, communication abilities, functional status, and trunk control. In contrast to our study, this research focused more on improving cognitive function and consciousness rather than on mobilization.

Other available studies about robotic mobilization therapy are limited to case reports: One demonstrated the use of VEMOTION^®^ with virtual reality in a critically ill individual [26]. Another showed improved active participation in VEMOTION^Ⓡ^ therapy over time, as evidenced by EMG recordings, hip range of motion, leg load force, and verticalization [17].

### Timing for early mobilization

Determining the optimal time frame to initiate early mobilization appears to be a crucial factor influencing both short- and long-term outcomes. Two landmark studies demonstrated that very early mobilization was not superior to standard care [8, 27]: The TEAM trial, which initiated mobilization at a median of 60 h after ICU admission, found no improvement in the number of days individuals were alive and out of the hospital [8]. Moreover, it reported an increased rate of AEs associated with the intervention. A study by Morris et al. [27] , in which mobilization began within less than one day, found no significant differences in median hospital length of stay, duration of mechanical ventilation or ICU care, or in physical and mental health scores at six months. In contrast, numerous studies have demonstrated the advantages of early mobilization over standard care [28–31]. A meta-analysis by Ding et al. demonstrated that early mobilization initiated within 72–96 h of mechanical ventilation was optimal for reducing ICU-acquired weakness [32]. Furthermore, initiation within 48–72 h of mechanical ventilation was associated with a greater reduction in the duration of mechanical ventilation. Another meta-analysis by Daum et al. [33] also found that mobilization within 72 h of ICU admission significantly reduced ICU and hospital length of stay and positively affected functional outcomes and quality of life. As our study focused on individuals in the IMC, most of whom had previously spent a considerable amount of time in the ICU, the intervention in our study began later, which limits direct comparability to the ICU-focused studies [34].

### Long-term results

In the study group, the Barthel Index improved significantly over time, whereas it remained stable in the control group, potentially indicating a long-term benefit of the add-on robotic therapy. However, these findings must be interpreted with caution. In both groups, the number of participants declined further due to deaths occurring after hospital discharge. Moreover, post-discharge rehabilitation varied substantially between individuals; in some cases, no rehabilitation was provided at all, which may also have influenced the observed outcomes. Notably, a higher proportion of participants in the study group were discharged directly to rehabilitation facilities compared to the control group. This difference in access to structured post-acute care may have placed the study group at a relative advantage regarding long-term functional outcomes, which should be considered when interpreting the follow-up results.

## Limitations

This was a pilot study involving a small sample from a single center. Large-scale multicenter trials are needed to validate these findings and enable their generalization to a broader patient group.

The unequal group sizes – with fewer individuals in the control group – may have led to an underrepresentation of the effects of standard physiotherapy. This imbalance was due to the relatively high dropout rate, which reflects the often unpredictable nature of clinical practice. In the context of the long-term results, further attrition occurred due to deaths following hospital discharge, resulting in an even smaller sample size. Therefore, these long-term findings must be interpreted with particular caution.

Moreover, participants had heterogeneous admission diagnoses, including both spinal and cranial involvement, and groups were not randomized for this. Among those with cranial injuries, the severity of traumatic brain injury varied considerably. This heterogeneity may have introduced bias and affected the comparability of outcomes.

For ethical reasons, the robotic therapy was implemented as an add-on intervention to ensure that standard physiotherapy, with its broader range of exercises and therapeutic modalities, was not withheld from the participants of the study group. However, this may have introduced a bias toward greater improvement in the study group, as these individuals received two therapy sessions per day.

As mentioned previously, post-discharge care pathways took place outside the controlled study setting and varied considerably between individuals. In the intervention group, 4 out of 7 participants (57.1%) were still undergoing rehabilitation at the time of the three-month follow-up interview and Barthel Index assessment. In the control group, 2 out of 5 participants (40%) were still in rehabilitation at that time. This difference in the level of ongoing care may have significantly influenced the three-month Barthel Index and must be considered a limitation of the study.

Another limitation of this study is that the physician responsible for participant screening, enrollment, and randomization was also involved in the interpretation of the results. Although this investigator was blinded to outcome assessment, complete blinding—particularly with regard to data analysis and interpretation—was not possible, which may have introduced some degree of bias.

This was not a blinded study, as blinding was not feasible due to the fundamental nature of the intervention. However, this also meant that the investigators assessing the CPAx and Barthel Index were not blinded to group allocation, which may have introduced bias in the evaluation of functional outcomes.

The focus of our study was not to evaluate robotic therapy as a means of process optimization or to reduce the physical workload of physiotherapists or nursing staff. Accordingly, no data were collected regarding the time or personnel required for the setup and dismantling of the robotic device, the physical and psychological burden on therapists or nursing staff associated with the use of robotic therapy, nor the extent to which daily ward routines were disrupted. These aspects should not be overlooked, even though robotic systems are designed with the intention of supporting users in every respect.

## Conclusion

The clinically significant improvements in the physical and respiratory function measured by the CPAx observed in the study group underline the positive impact of robotic mobilization as an adjunct to traditional physiotherapy. Furthermore, the therapy’s safety profile, with no AEs, SAEs, or disruptions related to the device, supports its feasibility in clinical practice. While our results are promising, further large-scale, multicenter studies are needed to confirm these findings and establish robotic mobilization therapy as a standard component of early mobilization therapy in individuals with critical illness.

## Supplementary Information

Supplementary Material 1.

## Data Availability

No datasets were generated or analysed during the current study.

## Abbreviations

AAN: Assist–as–Needed
ADL: Activities of Daily Living
AE: Adverse Event
BI: Barthel Index
BiPAP: Bilevel Positive Airway Pressure
CPAx: Chelsea Critical Care Physical Assessment Tool
CPAP: Continuous Positive Airway Pressure
EMG: Electromyography
EVD: External Ventricular Drain
HR: Heart Rate
ICU: Intensive Care Unit
IMC: Intermediate Care Unit
MAP: Mean Arterial Pressure
MRC: Medical Research Council
PaCO_2_: Partial Pressure of Carbon Dioxide
PaO_2_: Partial Pressure of Oxygen
RRsys: Systolic Blood Pressure
SAE: Serious Adverse Event
SpO_2_: Peripheral Capillary Oxygen Saturation
