# Supplementary material for: Safety and efficacy of add-on robotic therapy for early mobilization in intermediate neurocritical care: a pilot study
Source: J Neuroeng Rehabil. 2025 Oct 3;22:208. doi: 10.1186/s12984-025-01750-5 (PMC12492941; doi:10.1186/s12984-025-01750-5)
Supplement: Supplementary file 1 — Supplementary Material 1. [file 12984_2025_1750_MOESM1_ESM.docx]

**Supplementary Table 1: Adverse Events (AEs) and Serious Adverse Events (SAEs)**

| **Adverse Events (AEs)** | systolic blood pressure (RRsys) > 180 mmHg / < 80 mmHg  SpO2 < 90%  heart rate (HR) > 130 bpm / < 60 bpm  HR increase > 20%  new arrhythmias  respiratory rate > 30/min / < 10/min  requirement for resuscitation  deterioration in vigilance/agitation/(suspected) pain (Numeric Rating Scale > 3 or Behavioral Pain Scale > 6)  dislocation of access points/catheters/drains  vomiting/reflux  skin reactions to straps |
| --- | --- |
| **Serious Adverse Events (SAEs)** | death  cardiopulmonary instability requiring immediate intervention, such as intubation, administration of catecholamines, or cardiopulmonary resuscitation  transfer to the intensive care unit  injuries caused by the robotic therapy that result in permanent disability (e.g., paralysis, limb fractures, amputations) or a prolonged hospital stay |

**Supplementary Table 2**: Diagnosis and Treatment

|  | **Admission diagnosis** | **Treatment** | **Discharge** |
| --- | --- | --- | --- |
| **Study Group** |  |  |  |
| No. 1 | Traumatic acute subdural hematoma  Epidural hematoma | Hematoma evacuation  VP-shunt | To rehabilitation |
| No. 5 | Obstructive hydrocephalus | EVD  VP-shunt | To rehabilitation |
| No. 6 | Unstable L4 fracture | Lumbar fusion surgery | To another hospital |
| No. 7 | Traumatic hemorrhagic contusion  Acute subdural hematoma | EVD  VP-shunt | To another hospital |
| No. 8 | Traumatic subarachnoid hemorrhage | EVD  VP-shunt | To rehabilitation |
| No. 10 | Traumatic acute subdural hematoma | Hematoma evacuation  EVD  VP-shunt | To rehabilitation |
| No. 11 | Traumatic hemorrhagic contusion  Acute subdural hematoma | Traumatic brain injury monitoring | To rehabilitation |
| No. 14 | Unstable C5/6 fracture (type B1)  Acute subdural hematoma | Cervicothoracic fusion surgery | To nursing home |
| No. 16 | Traumatic hemorrhagic contusion | Traumatic brain injury monitoring | To rehabilitation |
| No. 20 | Unstable C2/3 and C3/4 fracture (type B1)  Traumatic hemorrhagic contusion | EVD  Cervical fusion surgery | To rehabilitation |
| No. 23 | CSF leak after decompression and dural plasty for Chiari Malformation Type I with syringomyelia | Dural plasty of the craniocervical junction  EVD  VA-shunt | To rehabilitation |
| **Control group** |  |  |  |
| No. 2 | Spondylodiscitis (multifocal) | Fusion surgery  Antibiotic treatment | To home |
| No. 12 | Traumatic hemorrhagic contusion  Acute subdural hematoma | Traumatic brain injury monitoring  EVD | To rehabilitation |
| No. 15 | Acute subdural hematoma | Hematoma evacuation  EVD | To rehabilitation |
| No. 21 | Traumatic subarachnoid hemorrhage | Traumatic brain injury monitoring  Hematoma evacuation (twist-drill craniostomy with screw) | To rehabilitation |
| No. 24 | Rheumatic dens pannus with absolute stenosis at C1/2 | C1-2 fusion surgery + decompression | To nursing home |
| No. 27 | Absolute stenosis Th11/12 with myelopathy | Fusion surgery + decompression | To rehabilitation |
| No. 28 | Spondylodiscitis (lumbar) | Fusion surgery  Antibiotic treatment | To nursing home |

EVD= external ventricular drain, CSF = cerebrospinal fluid, VA=ventriculoatrial, VP=ventriculoperitoneal


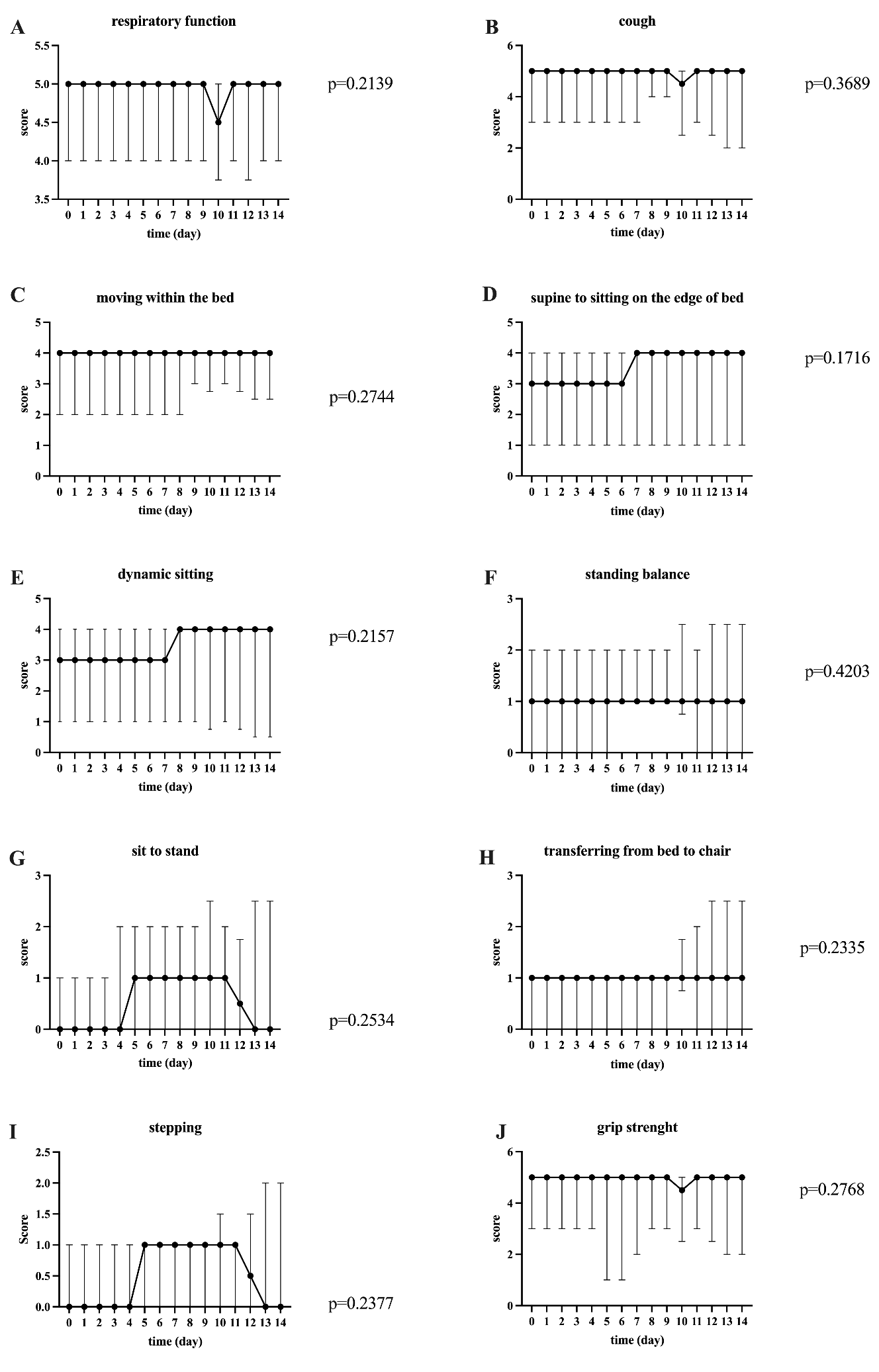


**Supplementary Figure 1 (A-J):** Supplemental Figure 1 A–J shows the daily median (IQR) score of the 7 individuals in the control group for each individual item of the CPAx. A mixed-effects analysis was performed for each item to test for significant changes over time.


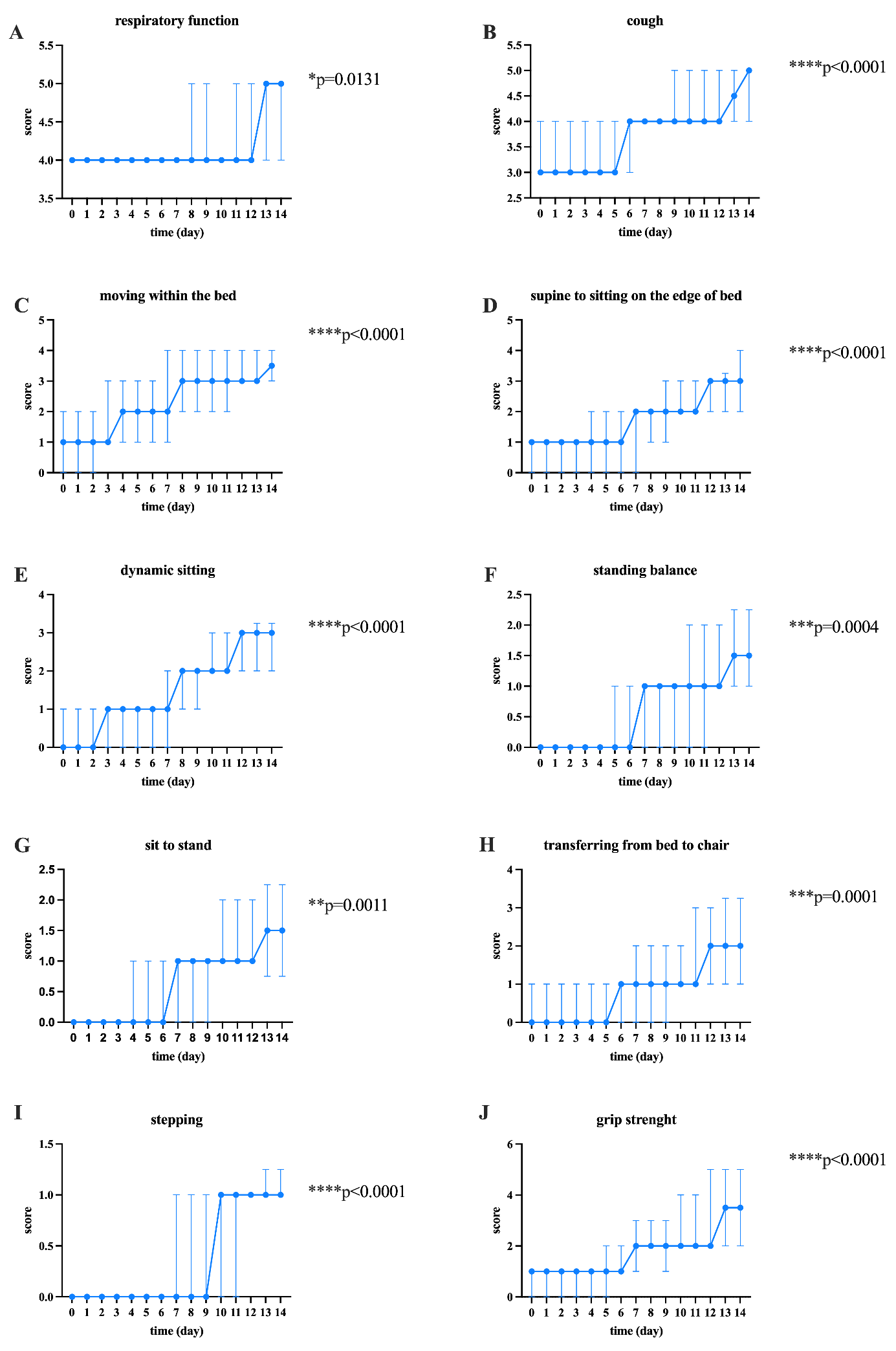


**Supplementary Figure 2 (A-J)** Supplemental Figure 2 A–J shows the daily median (IQR) score of the 11 individuals in the study group for each individual item of the CPAx. A mixed-effects analysis was performed for each item to test for significant changes over time.
